# Applications of Plant Bioactive Compounds as Replacers of Synthetic Additives in the Food Industry

**DOI:** 10.3390/foods13010047

**Published:** 2023-12-21

**Authors:** Gema Nieto, Lorena Martínez-Zamora, Rocío Peñalver, Fulgencio Marín-Iniesta, Amaury Taboada-Rodríguez, Antonio López-Gómez, Ginés Benito Martínez-Hernández

**Affiliations:** 1 Department of Food Technology, Nutrition and Food Science, Veterinary Faculty, University of Murcia, 30100 Murcia, Spain; gnieto@um.es (G.N.); lorena.martinez23@um.es (L.M.-Z.); rocio.penalver@um.es (R.P.); ataboada@agrosingularity.com (A.T.-R.); 2 Agrosingularity, Calle Pintor Aurelio Pérez 12, 30006 Murcia, Spain; 3 Food Safety and Refrigeration Engineering Group, Department of Agricultural Engineering, Universidad Politécnica de Cartagena, Paseo Alfonso XIII, 48, 30203 Cartagena, Spain; antonio.lopez@upct.es

**Keywords:** plant bioactives, natural antioxidants, natural antimicrobials, natural emulsifiers, natural colorants, natural flavorings, active packaging

## Abstract

According to the Codex Alimentarius, a food additive is any substance that is incorporated into a food solely for technological or organoleptic purposes during the production of that food. Food additives can be of synthetic or natural origin. Several scientific evidence (in vitro studies and epidemiological studies like the controversial Southampton study published in 2007) have pointed out that several synthetic additives may lead to health issues for consumers. In that sense, the actual consumer searches for “Clean Label” foods with ingredient lists clean of coded additives, which are rejected by the actual consumer, highlighting the need to distinguish synthetic and natural codded additives from the ingredient lists. However, this natural approach must focus on an integrated vision of the replacement of chemical substances from the food ingredients, food contact materials (packaging), and their application on the final product. Hence, natural plant alternatives are hereby presented, analyzing their potential success in replacing common synthetic emulsifiers, colorants, flavorings, inhibitors of quality-degrading enzymes, antimicrobials, and antioxidants. In addition, the need for a complete absence of chemical additive migration to the food is approached through the use of plant-origin bioactive compounds (e.g., plant essential oils) incorporated in active packaging.

## 1. Introduction

Substitution of chemical compounds from food products to obtain more natural food products is a target of worldwide interest, which must be understood as an integrated approach of three interconnected components: clean labels with natural additives, zero chemical packaging-to-food migrations through using systems such as active packaging with natural bioactive compounds, and an adequate integration in the final product (see also Graphical abstract). Synthetic food additives are substances that have been formulated or manipulated by a chemical process and altered chemically. Synthetic food additives have been conventionally used to extend the shelf life of food products by reducing oxidation processes (e.g., lipid oxidation, etc.), microbial growth, and ensuring food safety, among other quality-degrading processes. Hence, several synthetic antimicrobials (e.g., sulfites, benzoates, sorbates, sodium hypochlorite, etc.), antioxidants (e.g., nitrate, nitrite, butylated hydroxyanisole (BHA), tert-butyl hydroquinone, butylated hydroxytoluene (BHT), propyl gallate, etc.), synthetic colorants, and flavorings, among others, have been used. Nevertheless, several health problems have been associated with the consumption of these synthetic additives, as widely reported [1,2,3,4,5]. In that sense, the food industry is moving from synthetic additives to a strategy of natural additives [6], including bioactive compounds from plant products, such as fruits, vegetables, herbs, and spices. In addition, non-animal origin additives (mainly natural additives derived from plants) are in high demand by the food industry for vegetarian/vegan consumers.

These findings motivate the need for alternatives to synthetic additives, with the “Clean Label” approach for food products containing only natural additives from plant-origin that is exponentially gaining importance. In that line, the Food Naturalness Index (FNI) has also been proposed, which attempts to quantify the level of food naturalness precisely in the absence of clear guidelines [7]. The FNI concurrently incorporates and expands upon consumer research, legal, and technical viewpoints’ insights. In particular, the FNI comprises four component measures, namely farming practices, free from additives, free from unexpected ingredients, and degree of processing, which includes ten pertinent food naturalness attributes that can be reliably assessed from information on the product label. The degree of consumer perception of food naturalness was positively correlated with the FNI scores [7].

Plant extracts are considered GRAS (“generally recognized as safe”), which makes consumers and regulatory agencies regard them as more appropriate for use in food than synthetic compounds. Herbs and spices, and more especially their extracted essential oils (EOs), have aroused human interest for centuries due to their high antimicrobial and antioxidant properties. Accordingly, Egyptians used EOs between the bandages of their mummies because of their observed preservative properties. Currently, herbs, spices, and plant extracts are among the natural compounds that are being used as natural preservatives to extend the shelf life of food products, an alternative to conventional synthetic additives, due to their high antimicrobial, antioxidant, flavoring, colorant, and emulsifier properties, among others.

Plant products like aromatic herbs and spices are highly appreciated due to their culinary interest and have been used for centuries to improve the organoleptic properties of foods. The observed high antioxidant properties of these plant products were studied by Chipault et al. [8] in 1951, who reported that extracts from 32 different plants exhibited high antioxidant activity in different food products. There is currently a lot of information about the compounds of such plant extracts and their action mechanisms involved in the inhibition of quality degradation processes (e.g., lipid peroxidation) of food products.

The shelf life of food products can also be extended by the controlled release of active compounds (e.g., oxidants, antimicrobials, etc.) from active packaging. Active packaging consists of adding active compounds (with antimicrobial, antioxidant, or other preservative properties) into the packaging material to extend the product’s shelf life. The active substance is applied (usually as an encapsulated active substance) to the package surface, from which it is gradually released to the surrounding environment of the food product contained inside of this active packaging. For example, an excellent example of natural active packaging is cardboard packaging, including encapsulated (using cyclodextrins) EOs as a sustainable alternative to conventional petrochemical packaging [9,10]. Therefore, the use of plant EOs for active packaging is also a method to extend the product’s shelf life, reducing the use of synthetic additives.

Nowadays, the actual consumer profile is interested in food free from synthetic additives, including mainly natural additives. For example, natural extracts of the *Lamiaceae* family, such as rosemary, have been studied due to their high bioactive properties like hepatoprotective, antifungal, insecticide, antioxidant, and antibacterial. The biological properties of spices are mainly due to phenolic compounds. However, the use of spices or their plant extracts is limited because of their strong odor, color, and taste. In that sense, encapsulation of these compounds avoids their negative sensory effects due to their controlled release in low concentrations.

Owing to the new applications of natural extracts to extend the product’s shelf life, this review highlights the use of natural extracts from plants in food and food packaging due to their several properties to substitute synthetic additives aiming to obtain Clean Label products. Moreover, the relationship between their structure and related properties through their specific mechanisms is hereby reviewed. In addition, the extensive research in other health-promoting properties (apart from antimicrobial and antioxidant) of the bioactive substances from these plant products has gained a growing interest in industrial fields, apart from the food industry, like the cosmetic and pharmaceutical industries.

## 2. Synthetic Additives and Health Risks

The food industry typically employs synthetic additives as a cost-effective and efficient method to mitigate processes that degrade the quality of food products. These additives are categorized based on their functions, including preservatives, antioxidants, flavor enhancers, colorings, emulsifiers, stabilizers, gelling agents, thickeners, metal sequestrants, waxes, sweeteners, and products for treating flours and starch derivatives. Legislation imposes maximum usage limits on these additives due to potential toxic effects. Controversy surrounds certain additives like BHA, BHT, sulfites, and nitrates, which have been linked to significant health side effects, including asthma, hyperactivity, and cancer [11]. The main issues with these chemical additives are described in the following lines.

BHA (E-320) is a monophenolic antioxidant composed of two isomeric compounds: 2-tert-butyl-4-hydroxyanisole (90%) and 3-tert-butyl-4-hydroxyanisole (10%). Despite its efficiency in controlling oxidation in products with short-chain fatty acids, BHA has led to allergic reactions such as dermatitis due to extensive use [12]. BHT (E-321) is also a monophenolic antioxidant with lipophilic properties. Its efficacy is lower than BHA, attributed to the hindrance caused by two tert-butyl groups. While commonly used for oxidation protection in soybean oil and other materials, BHT has been associated with adverse reactions, including chronic urticaria [12]. Sulfites (E-220 to E-228) are widely used preservatives in the food industry, particularly in meat products, to maintain color and extend shelf life. Despite their benefits, sulfites, when metabolized inadequately, can cause harmful reactions, especially in individuals with enzymatic deficiencies, such as asthmatics [13]. Recent studies highlight the pro-oxidant and altering capacity of sulfites, potentially contributing to neurological dysfunction [14]. Nitrates, specifically nitrite, play a crucial role in preserving meat product colors. However, prolonged consumption poses risks, including acute toxicity and the formation of carcinogenic compounds like N-nitrosamines during cooking [15,16]. Admissible maximum doses are set to mitigate these risks [17].

In addition to them, other additive groups (emulsifiers, colorants, flavorings, etc.) are also linked to specific issues as described in the following lines. Most synthetic emulsifiers, such as phthalate esters, induce health problems and might cause toxic symptoms for consumers with long administration [18]. For example, male reproductive toxicities, dysfunction of enzymes, modification of protein structure, etc., together with other problems of global concern like biodegradability and aquatic toxicity, have been linked with some synthetic emulsifiers [19,20,21]. This is even more worrying, knowing some facts, such as that glycerin monostearate accounts for about 50% of the total emulsifier consumption in the food industry [19]. Several synthetic food colorants, such as azo-dyes, have also attracted the attention of national safety agencies due to their potential human toxicity [22]. For example, new toxicity concerns have arisen in several synthetic food colorants (e.g., Sunset Yellow, Tartrazine, Azorubine, Allura Red, Patent Blue V, etc.) due to their health issues such as the generation of carcinogenic metabolites, the ability to bind to human serum albumin, and hypersensitivity reactions, among others [22,23]. Synthetic flavorings are being questioned due to the long list of associated health problems, such as those related to monosodium glutamate, artificial sweeteners (aspartame, acesulfame K, and saccharin), hydrolyzed protein, etc. These synthetic flavorings have been linked as potential inducers of health issues like carcinogenicity, allergies, behavioral changes (anxiety, depression, insomnia, hyperactivity, attention deficit syndrome, and confusion), and infertility, among others (asthma, chest pains, nausea, bloating, burning sensations, carpel tunnel syndrome, etc.) [22].

Overall, the widespread use of synthetic additives raises concerns about serious health issues. Consequently, there is a growing interest in exploring natural alternatives, such as extracts and EOs from fruits, vegetables, herbs, and spices, for application in the food industry.

## 3. The Bioactive Properties of Natural Ingredients as Natural Additive Replacers of Synthetic Additives

In 2010, UNESCO acknowledged the term “Mediterranean Diet” as an Intangible Cultural Heritage of Humanity. This designation encompasses not only the dietary habits of individuals residing in olive-growing regions around the Mediterranean Sea but also their overall lifestyle, reflecting cultural, social, territorial, and environmental aspects [24]. Embracing this dietary pattern has been shown to offer potential health advantages, including cardiovascular and neuroprotective effects and antioxidant, anti-inflammatory, and anticarcinogenic properties [24]. These positive health impacts stem from the consumption of foods abundant in polyunsaturated fatty acids (found in fish), extra virgin olive oil, nuts, vitamins, minerals, and phenolic compounds from various herbs and spices, such as oregano, olive tree, rosemary, garlic, paprika, and a variety of fruits and vegetables.

Moreover, additives derived from plants in the Mediterranean Diet can serve as effective preventatives against the degradation of food components. For instance, these natural additives can mitigate lipid peroxidation by inhibiting chain initiation through the scavenging of radicals, breaking chain reactions, decomposing peroxides, reducing localized oxygen concentrations, and binding chain-initiating catalysts like metal ions. Consequently, the use of natural preservatives to extend the shelf life of products demonstrates comparable technological properties (e.g., antioxidant, antimicrobial. Emulsifiers, flavoring, colorants, etc.) to some synthetic additives. This promising approach is underscored by the diverse technological properties exhibited by numerous fruits (grapes, grape seed, pomegranate, date, and kinnow mandarin), vegetables (broccoli, potato, drumstick, and pumpkin), herbs (olive leaf, acerola, grape seed, cocoa, green coffee, and Ginkgo biloba), and spices (rosemary, green tea, black pepper, garlic, oregano, cinnamon, sage, thyme, mint, ginger, and clove) in various food products [25,26,27,28].

**Figure 1:**
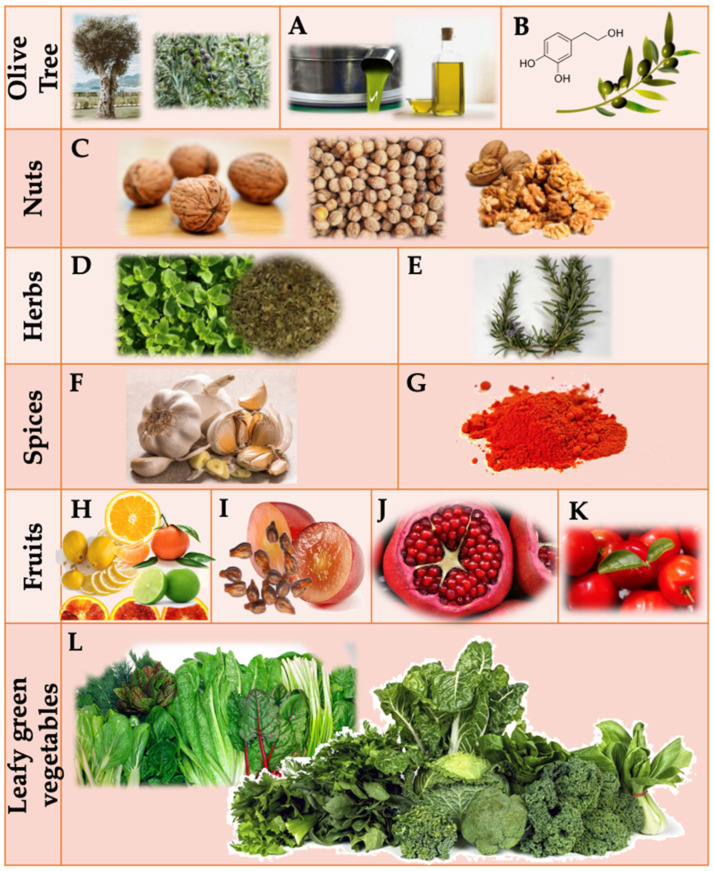
Ingredients as a source of natural extracts used as potential alternatives to synthetic additives: extra virgin olive oil (**A**), olive tree products rich in hydroxytyrosol (**B**), nuts (**C**), oregano (**D**), rosemary (**E**), garlic (**F**), paprika (**G**), citrus (**H**), grape seed (**I**), pomegranate (**J**), acerola (**K**), and green leafy vegetables (**L**).

Considering this, the present review focuses on the diverse technological properties—such as antioxidant, antimicrobial, emulsifier, flavoring, and colorant properties—of plant extracts. It also explores the associated action mechanisms, emphasizing their potential as natural food additives or for incorporation into active packaging. The discussion specifically delves into several Mediterranean plants and plant products, offering insights into their rich profiles of various natural additives, including hydroxytyrosol (HXT), nuts, extra virgin olive oil (EVOO), rosemary, pomegranate, grape seed, garlic, oregano, paprika, citrus fruit, acerola, and green leafy vegetables (Figure 1).

### 3.1. Hydroxytyrosol and Other Compounds from Olive Oil

One of the most potent natural antioxidant extracts found in the Mediterranean Diet is HXT, also known as 4-(2-dihydroxyphenyl)-ethanol, ranking just below gallic acid in strength. This compound exhibits antioxidant capabilities ten times greater than green tea and twice that of coenzyme Q10. Additionally, HXT’s scavenging ability rivals that of oleuropein and catechol. Obtained from olive leaf and oil, HXT contributes to the intense flavor and aroma of olive oil, with oleuropein being its precursor [29,30]. Studies by Merra et al. [31] and Lemonakis et al. [32] have demonstrated HXT’s high antioxidant capacity in vivo, reducing the risk of metabolic syndrome. With an additional hydroxyl group in its benzene ring compared to tyrosol, HXT functions as a more effective free radical scavenger, enhancing its antioxidant power, especially under stress conditions [32]. Extracts rich in HXT can vary in concentration widely among different olive varieties (0.02–600 mg kg^−1^) due to factors like growing geographical area, cultivation practices, harvest ripening stage, and postharvest processing [33]. These antioxidant compounds, rich in polyphenols, exhibit antimicrobial, antioxidant, anti-inflammatory, and anticancer properties, effectively reducing excess free radicals that can cause oxidative damage [34,35,36,37,38].

### 3.2. Nuts

Nuts are essential components of the Mediterranean Diet, comprising water, proteins, lipids (Ω-3 and Ω-6), carbohydrates, fiber, and an excellent lipid profile, along with antioxidant compounds. Oleic and α-linolenic fatty acids constitute 75% of total lipids, while saturated fatty acids remain below 7%. Nuts, a significant source of alpha-linolenic acid after oily fish, also contain antioxidants such as vitamin E (low levels of α-tocopherol, 20 mg kg^−1^, but high levels of γ-tocopherol, 450 mg kg^−1^), polyphenols, Se, Zn, Mg, and folic acid. Studies by Blomhoff et al. [39] and López Uriarte et al. [40] have shown that introducing tocopherols and polyphenols from nut extracts reduces in vitro lipid peroxidation and oxidative damage to animal DNA, enhancing antioxidant enzyme activity and decreasing cholesterol oxidation products. Additionally, Torabian et al. [41] observed a decrease in plasma oxidative biomarkers with the incorporation of nuts into meals.

### 3.3. Fruits and Green Leafy Vegetables

The Mediterranean Diet is globally recognized for its abundance of fresh fruits, with specific attention to grape, pomegranate, citrus fruit, and acerola. Grape seed EO, obtained from byproducts of wine production, stands out for its high content of therapeutic antioxidant compounds such as flavanols, ellagitannins, anthocyanins, and stilbenes [42]. Pomegranate extracts, particularly rich in punicalagin, among other phenolic compounds, offer antioxidant, anti-inflammatory, antibacterial, and anticancer effects [43]. Citrus fruits, including sweet and bitter oranges, contain bioactive compounds like naringin and hesperidin, acting as antioxidants by chelating iron and displaying hydroxyl group scavenging activity. Acerola, native to Central and South America, is a significant source of vitamin C, carotenoids, and bioflavonoids (anthocyanins and flavonols), enhancing its antioxidant potency [44].

Green leafy vegetables, widely consumed in the Mediterranean region, are rich in nitrates, offering potential alternatives to synthetic nitrates and nitrites in dry-cured food products. Varieties such as beet, lettuce, arugula, watercress, celery, spinach, and chard are studied for their natural nitrate content. Although nitrites can affect the human body adversely, green leafy vegetables, with their phenolic compounds (phenolic acids and flavonoids), can act as antioxidants along with nitrates [45,46].

### 3.4. Spices, Herbs, and Plant Essential Oils

Herbs and spices play a significant role in the traditional recipes of the Mediterranean Diet. Rosemary, a woody perennial herb from the region, is rich in phenolic compounds with anti-inflammatory, antioxidant, antiaging, antibacterial, and anticancer properties. Carnosic acid, carnosol, rosmarinic acid, and hesperidin are major components contributing to rosemary’s therapeutic attributes [47,48,49]. Oregano offers bioactive compounds divided into major components of oregano EO (such as thymol or trans-sabinene hydrate) and hydrophilic phenolic compounds (phenolic acids and flavonoids) [50]. Garlic is renowned for its high antioxidant and antimicrobial properties, primarily attributed to allicin, thiosulfinates, flavonoids, and ferulic acids [51]. Paprika, derived from red peppers, is a commonly used spice and natural colorant in cured sausages, providing aroma, color, flavor, and antioxidant power [52,53].

## 4. Natural Additives of Plant Origin as Alternatives to Synthetic Additives Classified According to Their Use

**Figure 2:**
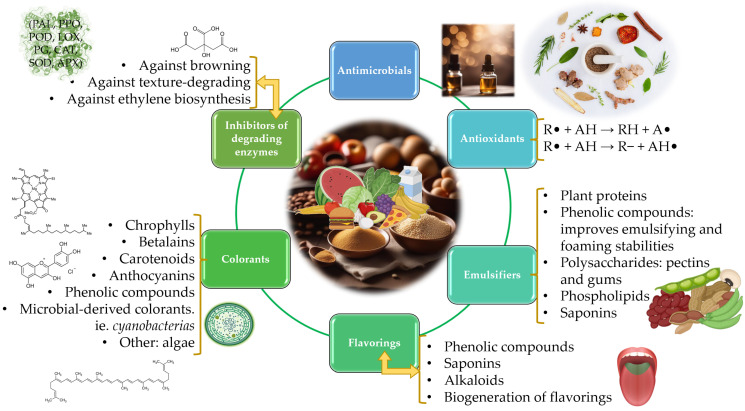
Natural extracts as alternatives to synthetic additives classified according to their use and function.

The natural additives of plant-origin alternatives studied in the present section are summarized in Figure 2.

### 4.1. Antimicrobials

The high antimicrobial effect of extracts of some herbs and spices makes them efficient and environmentally friendly alternatives to synthetic antimicrobial additives [54]. In addition, they are excellent active substances to include in active packaging with antimicrobial activity to extend the shelf life of food products. In particular, EOs extracted from plants can slow and retard microbial growth to increase the shelf life of the product [55]. EOs are aromatic liquids that are derived from various plant components (such as leaves, flowers, buds, etc.), and they have a complex composition made up of 20–60 distinct compounds with high antimicrobial properties, among other properties (antioxidants, etc.). Usually, two or three of these EO components account for up to 20–70% of the total EO composition. Typically, the factors affecting the main EOs’ biological or technological characteristics are those related to their major components [56]. The major EO component groups with high antimicrobial properties: (i) terpenes/terpenoids (e.g., carvacrol, thymol, etc.), which amount up to 90% of all major components of EOs, and ii) aromatic compounds (e.g., eugenol, cinnamaldehyde, etc.). These compounds possess the ability to disrupt the cells of microorganisms, although the mechanisms of action have not been elucidated. It is important to know that the antimicrobial activity of EOs depends on three characteristics: the hydrophobic or hydrophilic character, the synthetic components, and the type of microorganism to be inhibited [57]. Interestingly, some studies claim that EO vapors have a greater antimicrobial effect against the liquid form [58,59].

Gram-positive bacteria are more susceptible to such antimicrobial components of EOs than Gram-negative bacteria due to differences in cell wall composition. Cell shape also influences the susceptibility of rod-shaped bacteria, which are more vulnerable than cocci [60]. The antimicrobial activity of EOs is related to the lipophilic nature that allows them to accumulate in the membranes, where they act. They also degrade the bacterial cell wall, reducing the proton motive force and reducing intracellular adenosine triphosphate (ATP) levels [61]. The use of EOs as antimicrobial additives has been conventionally used in edible coatings [62]. Nevertheless, their high minimum inhibitory concentrations lead to undesirable characteristic EO-related flavors, which are not typical of the food product to which they are added. In addition, EOs are highly volatile and susceptible to oxidation processes due to light. For that reason, encapsulation of EOs allows a constant EO release of efficient concentrations of bacteriostatic effect, which is of high interest for antimicrobial active packaging [63].

Overall, several recent reviews have already addressed the antimicrobial properties of plant extracts, such as EOs, to be used as alternatives to conventional synthetic antimicrobials [55,64,65], so these additives are not reviewed on this occasion.

### 4.2. Antioxidants

Antioxidant compounds are substances that impede the oxidation of food products by either inhibiting the formation of free radicals or disrupting this process through specific mechanisms. Two primary pathways involve hydrogen atom transfer, where the antioxidant compound (AH) donates a hydrogen atom to a free radical (R•), creating a more stable radical (A•) (R• + AH → RH + A•), and electron transfer, where AH provides an electron to reduce the free radical (R• + AH → R- + AH•) [66]. Additionally, these compounds, based on their chemical nature and origin, may offer protection against bacterial development by inhibiting various functions such as cell wall maintenance, protein synthesis, transport, or DNA replication, serving as principal antimicrobial mechanisms. Nitrate and nitrite salts are employed in food products to control and prevent the growth of *Clostridium botulinum*. However, the consumption of nitrate/nitrite salts is regulated due to their natural presence in soil, vegetables, water, and animals, with levels increasing in recent years owing to nitrogen fertilizer use. To address this, utilizing natural sources of nitrates from green leafy vegetables could mitigate reliance on chemical nitrates and contribute to the development of food products free of synthetic additives [28].

Phenols and polyphenols, secondary metabolites found in plants, play essential roles in plant growth and development while serving as protective agents against pathogens. Structurally, they consist of an aromatic ring with one or multiple hydroxyl groups, influencing their organoleptic properties and antioxidant activity. Phenolic compounds, classified such as phenolic acids (hydroxybenzoic and hydroxycinnamic acids), phenolic diterpenes, flavonoids, and volatile oils, exhibit antioxidant capabilities through radical scavenging and metal chelating activities [66]. The classification of phenolic compounds is based on their structure and the presence of functional groups, as their antioxidant capacity relies on these factors.

The effectiveness of an antioxidant compound’s antioxidative properties is not solely determined by the number of hydroxyl groups (OH) but also by the arrangement of OH groups around the aromatic ring (ortho-, para-, and meta-). Numerous recent reviews [67,68,69] have explored the antioxidant properties of plant extracts as alternatives to conventional synthetic antioxidants, and therefore, these additives will not be reexamined in this context.

### 4.3. Emulsifiers

Emulsifiers are surface-active materials that are essential to the formation of emulsions because they facilitate emulsion formation and enhance emulsion stability. The choice of a proper emulsifier is one of the most important factors in the creation of successful emulsion-based products. In general, a wide range of artificial and natural emulsifiers, such as proteins, polysaccharides, phospholipids, and surfactants, can be used in the food industry [70]. The consumption of foods with emulsifying additives has increased greatly in recent years. Emulsifier food additives, especially chemically synthesized emulsifiers, have been the subject of extensive investigation in recent years due to preliminary evidence of adverse effects on gastrointestinal and metabolic health [71]. Therefore, research into new emulsifiers of natural origin based on bioactive plant products is necessary.

#### 4.3.1. Plant Proteins

Many proteins are excellent surfactants since the combination of hydrophilic and hydrophobic amino acids is found throughout their polypeptide chains. Hence, proteins stabilize food emulsions by decreasing the interfacial tension, electric repulsion, and steric hindrance, thus preventing droplet coalescence by making an encapsulating film around it [72]. The need to create plant-based proteins as natural food emulsifiers, with qualities similar to those of animal-origin proteins (caseins and whey proteins), is growing due to the “clean label” movement. The sources, structures, molecular weights, and adsorption behaviors of proteins all have a significant impact on the emulsifying qualities of food proteins [73]. Finding innovative processing methods that require little processing is essential for food technologists, as there is growing interest in using natural food emulsifiers to create “clean label” products. Finding commercially viable sources of protein, developing efficient techniques for protein separation, fractionation, and purification, and characterizing emulsifier functionality in terms of emulsion formation and stability are the three main challenges in this field. A particular kind of emulsion called Pickering emulsion stabilizes the emulsion by increasing the adsorption force on the surface of solid particles through physicochemical treatment and techniques like high-pressure emulsification. Although their processing method’s complexity and limited applicability are its drawbacks, recent techniques for stabilizing emulsions are being studied. The potential application in the food industry could be possible when a stable emulsion is formed with physical forces rather than chemical treatments.

Lentil protein, pea protein, faba bean protein, and soy protein are suitable candidates as emulsifiers [72]. Cereals are a cheap and abundant source of protein, but their limited ability to dissolve in water has limited their use as food emulsifiers. To improve these proteins’ suitability as emulsifiers, several researchers have chemically, enzymatically, and physically modified them [74]. Burgos-Díaz et al. [75] obtained Pickering oil-in-water emulsions with excellent stability and inhibited creaming using protein aggregate particles derived from lupin seeds. Lentil, pea, and faba bean protein concentrates were proposed as natural emulsifiers in oil-in-water algae emulsions fortified with omega-3 fatty acids produced by high-pressure homogenization [76]. By creating a physical barrier or functioning as an encapsulating film surrounding the oil droplet, proteins isolated from rice, peas, and potatoes demonstrated effective emulsion stabilization [77]. Similarly, whey protein, gum arabic, high-methoxy pectin, and soy protein isolate were used to create water–oil–water emulsions. Good release, stability, and manufacturing qualities were demonstrated by these emulsions [78]. Zhang et al. [79] found that plant-based emulsifiers obtained from almond protein isolates and camellia saponins reduced the oxidation of proteins and lipids as well as enhanced the physical stability of walnut oil-in-water emulsions. The ability of colloidal complexes made from pea proteins and grape seed proanthocyanidin to produce stable oil-in-water emulsions was also reported [80]. Pea and soy protein extracts were used as stabilizers of encapsulated orange EO [81]. Furthermore, Shao and Tang (2016) showed that pea protein can be used as a potential Pickering stabilizer, with varied stabilization processes according to oil ratio. They produced gel-like Pickering emulsion with diverse oil fractions using pea protein in an acidic setting (pH 3.0). Peanut protein extracted by alkali and acid precipitation was proposed as a food-grade Pickering stabilizer [82]. Rice glutelin (protein hydrolyzed using trypsin) improved the stability of lipid droplets, thus acting as a natural emulsifier in the development of label-friendly emulsion-based food products [83]. Soy protein concentrate was used for the preparation of rice bran oil-based lycopene emulsions since it improved the stability of lycopene in oil-in-water emulsions [84]. Pumpkin seed protein isolate (obtained by alkali extraction with isoelectric precipitation) was also proposed as a food emulsifier [85].

Apart from the above-described techniques, other approaches like chemical or mechanical disintegration and solvent-induced techniques have been investigated for using different protein sources as Pickering stabilizers. Specifically, the application of sodium or calcium ions may cause protein agglomeration, even while Pickering stabilization cannot be achieved due to insufficient strength of the primary hydrophobic contact [73]. Heat-treated soy glycinin nanoparticles demonstrated increased surface hydrophobicity and particle size, resulting in the formation of a stable emulsion by creaming and coalescence [86].

#### 4.3.2. Phenolic Compounds

Natural phenolic compounds (tannic acid, gallic acid, (-)-epigallocatechin gallate, and epigallocatechin) were applied as natural modifiers of the structural and functional properties of myofibrillar protein based upon their molecular interactions with proteins [87]. Tannic acid, epigallocatechin gallate, quercetin, and quercitrin also greatly improved the emulsifying and foaming stabilities of casein [88]. Flaxseed protein isolate was complexed with phenolic compounds (flaxseed polyphenols and HXT), showing the flaxseed protein isolate-stabilized emulsion (with higher charge density) and higher physical stability [89]. Porcine plasma protein hydrolysates, modified with oxidized tannic acid or oxidized chlorogenic acid, induced a higher emulsifying stability index, being proposed as potential emulsifiers in emulsion food systems [90].

#### 4.3.3. Polysaccharides

Certain hydrophilic carbohydrate chains in natural polysaccharides already have non-polar groups or proteins attached to them, which gives them good emulsifying properties. These kinds of surface-active polysaccharides are most frequently found in gum arabic, pectin, and galactomannans. Gum arabic is currently the natural polysaccharide-based emulsifier that is used in the food industry the most, especially in beverage emulsions. The main drawback of gum arabic, however, is that stable emulsions need a relatively high emulsifier-to-oil ratio (1:1) [70].

Gum arabic and pectin were examined by Liu et al. [78] as possible hydrophilic emulsifiers for the formation of water–oil–water emulsions alternative to whey and soy protein isolates. They found pectin superior to gum arabic, whey protein isolate, and soy protein isolate in its ability to create double emulsions with small droplets. This could be attributed to its superior ability to increase the viscosity of the emulsions, which in turn increased the strength of the shear forces produced during homogenization.

Citrus pectins can also be used as emulsifiers, according to recent research, with the degree of methoxylation and molecular weight determining how well they form and stabilize emulsions [91]. Additional research has demonstrated the effectiveness of polysaccharides extracted from basil seeds as emulsifiers, and the presence of protein moieties and non-polar groups on the carbohydrate backbone is thought to be responsible for surface activity [92]. Due to the presence of protein moieties, corn fiber gum has also been demonstrated to have good emulsifying properties [93,94]. Similar to proteins, more research is required to economically identify, isolate, and characterize the properties of polysaccharide-based emulsifiers derived from natural sources [95].

#### 4.3.4. Phospholipids

Phospholipids are natural amphiphilic molecules present in the cell membranes of microbial, plant, and animal species. These phospholipids, which are usually referred to as lecithin, can be separated, refined, and used as emulsifiers in the food industry. Lecithin is typically extracted from soybeans, egg yolks, milk, sunflower kernels, or rapeseeds [96]. Phospholipids form interfacial layers that are prone to coalescence, which makes them poor emulsifiers even though they are surface-active [97]. Nonetheless, depending on the combination of phospholipids they contain, some forms of lecithin do seem to be useful at forming and stabilizing emulsions [96].

#### 4.3.5. Saponins

Saponins are small water-soluble amphiphilic molecules that can be isolated from a variety of natural sources [98]. Because saponins have hydrophilic sugar groups joined to non-polar aglycone groups, they have surface activity. A food-grade ingredient named Q-Naturale, which is made of saponins extracted from the bark of the *Quillaja saponaria* tree, has been recently commercialized [99]. These quillaja saponins are expected to have a high potential in the food industry because of their demonstrated ability to form emulsions with small droplets that are stable to a variety of environmental stresses [100,101].

### 4.4. Flavorings

Plants produce flavor molecules either as a defense mechanism or from flavor precursors (such as amino acids, fatty acids, and carbohydrates) during harvesting, processing, and storage. It is very challenging to cover every compound that affects flavor because of how widely distributed their flavor thresholds are and how small variations can have a big influence. Furthermore, the presence of other components, as well as the matrix’s physical and chemical characteristics, influence how flavors are perceived. Focusing on active-flavor volatile compounds, Wang et al. [102] reviewed the main flavor descriptions and volatile profiles of several plant ingredients of interest (soy, pea, faba bean, chickpea, cowpea, lentil, lupin, adzuki bean, black bean, green bean, flaxseed, hemp, kidney bean, mung bean, navy bean, and pinto bean). Carboxylic acids, aldehydes, alkanes, alkenes, alkynes, alcohols, furans, ketones, esters, lactones, acetates, aromatic compounds, terpenes, sulfuric, and amino compounds were among the groups of compounds identified. The most prevalent compounds were alcohols and aldehydes. Among these, it was observed that a variety of protein-rich materials contain hexanal, octanal, nonanal, 1-hexanol, 1-octen-3-ol, and octanol. It is important to remember that different volatile compounds have different effects on flavor. Hexanal, 1-octen-3-ol, 1-hexanol, and 2-pentylfuran, for example, are lipid oxidation products linked to disagreeable smells. However, limonene, which is present in a variety of plant-based ingredients, is typically associated with pleasant citrus scents. Furthermore, it is important to keep in mind that different volatile compounds have different odor thresholds, which means that a compound’s perception of its presence depends on its concentration.

The non-volatile taste-active compounds found in plant ingredients, particularly those derived from legumes, include phenolic compounds (p.e., phenolic acids, tannins, and phenolic acids), saponins, and alkaloids. It has been discovered that these substances contribute to astringency, acrid (or pungent) flavor, and bitter taste.

#### 4.4.1. Phenolic Compounds

They exhibit distinctive flavors (lower molecular-weight aromatic phenolics, such as vanillin, eugenol, and vinylphenol) and astringent and bitter-tasting tannins [103]. Phenolics can be present in plants in three different forms: free and soluble, esterified or conjugated (to sugars and low-molecular-weight compounds), and bound and insoluble. Compared to insoluble-bound phenolics, which are covalently bound to structural elements of the cell wall, free and esterified phenolics are water-soluble and more flavor active (e.g., bind to taste receptor cells) [104]. Among them, the most interesting due to their characteristic flavors to be used as natural flavorings in foods are flavonoids, phenolic acids, and tannins.

*Phenolic acids* are found in high concentrations in the cotyledons of legume seeds. They can be classified as hydroxycinnamic acid derivatives (e.g., ferulic, p-coumaric, caffeic, chlorogenic, and sinapic acids [105]) and hydroxybenzoic acid derivatives (e.g., gallic, p-hydroxybenzoic, protocatechuic, vanillic, and syringic acids), with a C6–C1 structure. Beans, peas, and lentils contain primarily free and/or esterified phenolic acids, namely protocatechuic, hydroxybenzoic, gallic, ferulic, and p-coumaric acids. The complex sensations of sourness, astringency, and bitterness evoked by phenolic acids are dependent on the acid’s structure and concentration. For example, Langfried [106] described that ferulic acid was more bitter than vanillic acid, and vanillic acid was perceived as a sourer. A special mention is vanillin and its isomers, which is the fifth most consumed aroma compound (after 2-phenylethyl alcohol and esters, benzyl acetate, geraniol, and esters and ionones) in the world in flavor and fragrance compositions [107]. In particular, the vanillin isomer 2-hydroxy-4-methoxybenzaldehyde is typically found in the rhizomes and roots of medicinal plants, and, apart from their medicinal uses, it is used as a food flavoring. Vanillin can be naturally extracted from vanilla beans, but since the beans are pricey, the compound is also obtained from lignin waste, eugenol (an ingredient that is easily obtained), or guaiacol.

*Flavonoids* are the main phenolic substances found in grain legumes. Flavonols, flavones, flavanones, isoflavones, anthocyanidins, and flavanols are the structural subclassifications for them. Flavonoids have a variety of tastes, including bitter (most subclasses; Ref. [108]), astringent (flavanols), and sweet (dihydrochalcones). The type of interlinkage and degree of polymerization of flavonoids determine how strong their bitter and astringent properties are. Compared to dimers and trimers, flavanol monomers (such as catechins) typically have a higher astringent and less bitter quality [109].

*Tannins* are mainly found in plants and plant-based diets as condensed tannins. The astringency brought on by tannins is due to interactions between tannin and the proline-rich proteins in the saliva and adsorption on the cells of the oral mucosa. Tannins occur naturally at concentrations of 0.3 to 24 g (catechin equivalent) kg^−1^ in the test (seed coat) of a variety of beans and peas, including soy, faba bean, chickpea, pea, kidney bean, and cowpea [110,111].

#### 4.4.2. Saponins

Legume seeds contain a high content of saponins, which are secondary plant metabolites. Saponins have been widely classified as antinutrients, but more recently, their positive effects—such as their hypocholesterolemic, antioxidant, and anticancer effects in humans—have been investigated [112]. The highest concentrations of saponins found in legumes can be found in the seeds of soy, chickpeas, faba beans, and peas; their saponin content ranges from 25 to 56 g kg^−1^ [112]. The intensity of the bitter taste of saponins is associated with their chemical structure and appears to be concentration-dependent. For instance, DDMP (2,3-dihydro-2,5-dihydroxy-6-methyl-4H-pyran-4-one)-conjugated saponin from pea seeds has exhibited a significantly higher bitterness level and a lower odor threshold than saponin B (perceived at <2 and 8 mg mL^−1^, respectively; Ref. [113]). When the concentration of pea saponin extracts was increased from 2 to 12 mg mL^−1^, its perceived bitterness gradually increased [113].

#### 4.4.3. Alkaloids

Alkaloids are a broad and varied class of heterocyclic compounds with one or more nitrogen atoms. The majority of alkaloids have a bitter taste, including quinine (e.g., used in tonic water, usually at ≈80 mg L^−1^) and caffeine (e.g., used in cola-type drinks at ≈100 mg L^−1^).

#### 4.4.4. Biogeneration of Flavorings

Interestingly, smart combinations of biocatalytic and thermal steps have also been proposed as biomediated generation of food flavors aiming at sustainable flavor production inspired by nature, as previously reviewed [114]. For example, flavor generation is conducted based on fermentation, engineered microbial cells, isolated enzymes, and cell-free bioprocessing techniques. These approaches provide a cost-effective, efficient, sustainable, and clean method of producing flavors. Hence, targeted bioconversion of particular raw materials and ingredients should ideally release the flavor precursors needed for the proper thermal processing to produce the desired flavor profile. In industrial food processing, the blending of heat-induced food processes (such as drying, extrusion, and roasting) with fermentation or enzymatic treatment of raw materials represents a sophisticated method for producing flavors under gentle conditions. Traditional cooking methods and minimal processing conditions—strategies inspired by nature—have emerged as appealing ways to produce real flavor profiles that satisfy consumers’ desire for greater naturalness [114].

### 4.5. Colorants

Food manufacturers are using more synthetic food coloring than natural food coloring to achieve particular goals including homogeneity, high color intensity, low cost, and better looks. However, the “Southampton study” found a possible link between children’s increased aversion to attention deficit hyperactivity disorder and their drinking of beverages that include benzoic acid and azo dyes [5]. As a result, from July 2010, it has been required to label food colored with synthetic dyes with warning notices. This has led to a steady increase in the use of natural food coloring. Therefore, natural colorants are safer, more environmentally friendly, in harmony with nature, derived from renewable resources, and require less chance of chemical reactions during production. They may also be disposed of.

Water or diluted alcohol has traditionally been employed to extract natural colors that are soluble in water, while non-polar solvents are utilized to extract lipophilic pigments [115]. Hexane (carotenoids from Gac fruit peel), acetone (lycopene from tomato pulp waste), methanol (anthocyanins from eggplant peel), and trifluoracetic acid (betalains from papaya fruit peel) are a few examples of the non-polar solvents used for pigment extraction that are primarily of petrochemical origin [115]. Solvents such as glycerol, water, ethanol, ionic liquids, and fruit and vegetable oils (soybean, rapeseed, and cocoa oils, among others) are used in greener extraction procedures. These greener extraction techniques are assisted with innovative and extraction-efficient techniques like ultra-sounds, microwaves, pressurized liquids, supercritical fluid pulse-electric fields, and enzymatic extractions [115]. In addition, green extraction techniques are being implemented with nanotechnology to enrich the bioavailability and stability of extracted natural colorants, the process efficiency dependent on the used encapsulating material, nature of the encapsulant, encapsulation methods, nature of the core (natural pigment) material, and other processing conditions.

As described in the next sections, major plant pigments include chlorophyll (green), carotenoids (yellow, red, and orange), and flavonoids: anthocyanins, anthoxanthins (red, blue, and purple), and betalains (red, yellow, and purple). Other natural colorants proposed are also polyphenols.

#### 4.5.1. Chlorophyll

Chlorophylls are oil soluble and the most widespread distributed pigments responsible for the characteristic green color of plants [116]. The main chlorophylls in foods are chlorophyll a (intense blue-green) and chlorophyll b (dull yellow-green). Since acids and enzymes are the main variables influencing the breakdown of chlorophylls, deactivating the enzymes would increase the stability of the pigments (e.g., blanching) or prevent an acidic condition.

#### 4.5.2. Betalains

The majority of plants in the order Caryophyllales contain betalains, which are nitrogen-containing, water-soluble plant pigments. In addition to edible plant components, betalains are also found in flowers, leaves, stems, and bracts. These are divided into two groups: the yellow betaxanthins (found in *Mirabilis jalapa*, *Portulaca grandiflora*, *Opuntia ficus-indica*, and *Stenocereus pruinosus*) and the violet betacyanins (mostly extracted and found in beetroot, *Amaranthus caudatus*, *Gomphrena globosa*, and *Bougainvillaea glabra*). Betanin, or betanidin-5-O-glycoside, is the most prevalent betacyanin and the main pigment found in red beetroot roots [117]. At pH 4–5, betanin is red or dark red. As pH rises, it becomes bright bluish-red or blue-violet, and at alkaline pH levels, it finally turns yellow-brown. Because betalains are quite stable over the wide pH range of 3 to 6, they can be used in foods that are not very acidic [116]. Antioxidants such as ascorbic acid, iso-ascorbic acid, citric acid, and other preservatives can stabilize betalains to a certain degree, while antioxidant phenolic compounds have no stabilizing impact on the pigments [118]. Blanching is typically used to inactivate the betacyanin decolorizing enzyme, which tends to cause color fading, to increase the stability of betalains [119]. When β-cyclodextrin was added, betalain stability increased, most likely as a result of a 1:1 inclusion complex forming [120].

#### 4.5.3. Carotenoids

Plant pigments called carotenoids have 40 carbon atoms in each molecule. Because the body can convert carotenoids into retinol, they contain provitamin A activity [121]. All higher plants and certain animals have lipid-soluble pigments called carotenoids, which are yellow-orange-red. The majority of plant-based carotenoids are trans-isomers, and during food processing, these trans-isomers isomerize to cis-isomers [122]. Plant pigments’ redness and yellowness tend to decrease as a result of oxidation and isomerization processes, which are the primary causes of carotenoids’ degradation [123]. α-carotene is obtained from Buriti, carrot, *Cucurbita moschata*, and red palm oil. β-carotene is present in carrot, acerola, apricot, bocaiava (*Acronomia makayliyba*), broccoli, Buriti, cantaloupe, carrot, *Cucurbita maxima*, *Cucurbita moschata*, green leafy vegetables, mamey, mango, peach palm (*Bactris gasipaes*), pink grapefruit, red palm oil, red pepper, yellow and orangefleshed sweet potato, and tucum (*Astrocayum vulgare*). β -cryptoxanthin is found in Caja (*Spondias lutea*), pitanga (*Eugenia uniflora*), red pepper, and tamarillo. Plants rich in lycopene are pink grapefruit, pink-fleshed guava, red-fleshed papaya, pitanga (*Eugenia unijora*), tomato, watermelon, and tomato. Lutein is found in green leafy vegetables, broccoli, brussels sprouts, corn, and *Cucurbita maxima*. Buriti and corn are rich in zeaxanthin. Bixin and norbixin are apocarotenoids of yellow-to-red color extracted from the spiny seed pods of *Bixa orellana* L. (also known as annatto or achiote) [3]. Capsanthin and capsorubin are carotenoids responsible for paprika’s yellow-to-orange and red colors, respectively, from which paprika oleoresin is obtained, a purified form of the coloring compounds [3]. Crocin is the intense yellow pigment found in the spice saffron, which is the dried stigmas of the flowers of the crocus bulb, *Crocus sativus* (saffron crocus). Crocin, along with other crocetin glycosides, is found in the fruit of *Gardenia jasminoides* and *Gardenia augusta* [124].

#### 4.5.4. Anthocyanins

Plant pigments called anthocyanins are soluble in water. Many fruits and vegetables have blue, purple, red, and orange colors because of them [125] (Table 1). They are sensitive to pH changes, turning blue as the pH rises and reddest in extremely acidic environments [116]. Therefore, pH, light, temperature, and structure all affect the color and stability of these pigments [116]. To get the most stable colorant, the source of anthocyanins must be taken into account in addition to making the appropriate pH adjustment. Because of their acylation, anthocyanins from red sweet potato, black carrot, red radish, and red cabbage are said to be more resistant to heat and pH changes than anthocyanins from other sources. Red wine byproducts, mainly cyanidin, peonidin, malvidin, delphinidin, and petunidin, are the most important sources of anthocyanins for the food industry [3]. Resveratrol has also gained great importance due to its good health benefits [126].

#### 4.5.5. Phenolic Compounds

The most extensively studied substances include flavanones (naringin), flavones (4’,5,7-trihydroxyflavones and apigenin), and flavonols (fisetin, myricetin, myricitin, quercetin, and rutin); nevertheless, most applications are currently with commercial goods. The only plant-derived substances were myricetin and myricitrin, which were separated from the roots of *Myrica cerifera* L. [127].

The quercetin aglycone has a crystal form with a brilliant yellow color and is poorly water-soluble. However, its colorant capacity has been little exploited by the food industry [128]. Apigenin is a yellow crystalline powder insoluble in water, being the aglycone of several naturally occurring glycosides. It is present mainly in flowers, where one of the most common sources is chamomile, and in lower levels in some fruits (guava, bilimbi, and sweet cherry) and vegetables (bell pepper) [128]. The characteristic yellowish color of cider and apple juices is thought to result from the oxidation of dihydrochalcones like phlorizin. Because of this, the oxidized phlorizin product made from apples has also been suggested as a natural yellow pigment substitute for curcumin, which is poorly soluble in water, and tartrazine, which is linked to attention deficit hyperactivity disorder. It has a bright yellow color at Ph < 5 and an orange to reddish hue at pH > 6 [129]. Lately, Fromm et al. [130] proposed a green production process of oxidized phlorizin (instead of chemical-based oxidation processes) using enzymatic oxidation of apple seed extracts using a mushroom polyphenoloxidase.

Polyphenols can be extracted from grape pomace using green extraction procedures free of sulfites, such as the process proposed by Kammerer et al. [131] with enzymatic extraction using pectinolytic and cellulolytic enzyme preparations. The yields obtained by that proposed procedure were comparable to those from extractions applying sulfite. Other polyphenol extraction procedures from grape pomace have also been proposed, like dynamic superheated liquid extraction of phenolics, subcritical water/pressurized hot water/pressurized solvent extraction or semicontinuous hot–cold extraction, supercritical fluid extraction, the application of high voltage electrical discharges, microwave-assisted extraction, and extraction assisted by pulsed ohmic heating (as compiled by Kammerer et al. [132]). Nevertheless, scaling up such sophisticated extraction methods will be challenging due to cost-intensive equipment and safety precautions [132].

Curcumin (E100) is a diarylheptanoid, belonging to the group of curcuminoids, which are phenolic pigments responsible for the yellow color of turmeric, a spice traditionally used to color and flavor several foods ([133]). The beneficial health properties of curcuminoids have been increasingly reported in the late years [133,134].

#### 4.5.6. Microbial-Derived Colorants

Microorganisms that produce natural colorants are a highly sought-after field. Specifically, the genus Monascus produces monascin, a secondary yellow natural pigment. Aside from their intriguing properties as food coloring, there is no established consensus over their safe usage, and there is no E code. In contrast, their use is forbidden in Germany and many Asian countries, but they are widely used in food products [135]. c-phycocyanin is a blue pigment produced from *Arthrospira platensis* (cyanobacteria) [136].

#### 4.5.7. Other Natural Colorants Extracted from Plants

c-phycoerythrin, a reddish-orange pigment derived from blue-green algae [137], the freeze- and hot-dried aerial parts of *Crithmum maritimum* L., give food items like pasta, sauces, rice, fish, and meat a fascinating grey and green hue, respectively [138]. When added to drinks, juices, nectars, desserts, and gels, genipin—a blue pigment derived from the fruits of *Gardenia jasminoides* Ellis and *Genipa americana* L.—also demonstrated notable and attractive potentialities [139,140,141]. The red pigment known as madder, which is extracted from the roots of *Rubia tinctorum* L., has also been shown to increase the acceptance of hams, sausages, cooked fish, paste, drinks, and even some confectionary items, as well as the violet pigment violacein isolated from the bacteria *Chromobacterium violaceum* UTM 5 when added to yogurt and jelly [142]. Regarding natural pigments of brown colors (mainly obtained from sugar caramelization processes), toasted partially defatted cooked cottonseed flour produces natural pigments of light to dark brown. Black colors, apart from the vegetable carbon black, can also be obtained from animal origin, like squid ink, which is traditionally used as a food ingredient for some pasta [143].

### 4.6. Inhibition of Activity of Quality-Degrading Enzymes

Extracts from plants may also exert inhibitory effects on the activity of enzymes that cause degradative reactions on food products, which reduce their shelf life and consequently may compromise their food safety. Hence, several plant extracts have shown inhibitory effects against browning enzymes (polyphenol oxidase (PPO), peroxidase (POD), phenylalanine ammonia-lyase (PAL), etc.), texture-degrading enzymes (pectinesterases (PE), polygalacturonase (PG), etc.), oxidation of lipid compounds (lipoxygenase (LOX)), etc.

#### 4.6.1. Properties against Browning Enzymes

The mechanism of EOs to inhibit the activity of browning enzymes has been hypothesized to be the result of a competitive interaction of the EO components with the activity of these enzymes against the enzyme binding to their substrates [144]. Hence, Chen et al. [144] reported that eugenol and clove EOs inhibited the activities of the browning enzymes PAL, PPO, and POD in fresh-cut lettuce [144].

Orange oil nanoencapsulated into chitosan nanoparticles showed an inhibitory effect on the PPO activity, together with an antimicrobial effect [145]. Taheri et al. [146] reported that nanoencapsulated EOs from Iranian golpar (*Heracleum persicum*) reduced the PPO activity of bell peppers packaged with active paper, including these active compounds. Similarly, nanoencapsulated Iranian golpar EO showed higher antioxidant activity than synthetic additives (tert-butylhydroquinone) when added to soybean oil [147]. Another study observed the ability of eugenol (the major component of clove and other spices) to inhibit browning enzymes like POD, PPO, and PAL in fresh-cut Chinese water chestnut (*Eleocharis dulcis*), with PAL being the main enzyme inhibiting browning [148]. The activity of PPO and LOX was also decreased with ginger and angelica EOs, preventing the deterioration of the cell membrane structure and function [149].

#### 4.6.2. Properties against Texture-Degrading Enzymes

Other enzymes like PG and pectin methylesterase, which are involved in maintaining the integrity of plant cells, were inhibited in flat peaches when packaged using an active paper with nanoencapsulated EOs [150]. Similarly, the addition of basil EO reduced the activity of PG and other cell wall-degrading enzymes jamun fruit (*Syzgium cumini* L.) [151].

#### 4.6.3. Properties against Ethylene Biosynthesis Key Enzymes

Ethylene is considered the ripening hormone in plant products, so their biosynthesis may be reduced during the product’s shelf life. The ethylene biosynthesis pathway starts with the conversion of S-adenosyl-L-methionine into l-aminocyclopropane-1-carboxylic acid (ACC), being considered key enzymes for the ACC oxidase (ACO) and ACC synthase (ACS) [152,153]. Several solutions have been put forth to lessen the impacts of ethylene and can be divided into two categories: (i) lowering the amount of ethylene produced by the plant product and (ii) removing the produced ethylene from the atmosphere around the plant product [154]. However, the majority of these methods are expensive and/or rely on chemical products, which the average consumer may find objectionable since they prefer more natural goods made without chemical synthesis additives [155]. Surprisingly, EOs have a strong potential to lower the postharvest biosynthesis of ethylene in plant products. Particularly, high inhibition of ACO and ACS activities, and consequently lower ethylene production, was observed in fruit (blackberries and blueberries) and vegetables (tomatoes and broccoli) using active packaging with encapsulated EOs [156,157].

Extracts from plants have also found the ability to enhance the activity of endogenous antioxidant enzymes. For example, the activities of superoxide dismutase (SOD) and catalase (CAT) in sweet peppers increased when treated with an edible coating enriched with cinnamon EO [158]. Ascorbate peroxidase (APX) and SOD activities of mushrooms also increased when treated with an edible coating, including nanoencapsulated cumin EO [75]. The activities of the antioxidant enzymes CAT, SOD, APX, and glutathione reductase (GR) were also enhanced in shiitake mushrooms with vapor treatments of clove EOs, cinnamaldehyde and thymol [159]. Nanoencapsulated EOs from Iranian golpar (*Heracleum persicum*) also enhanced the SOD and CAT activity of bell peppers [146].

The activities of the antioxidant enzymes GR and APX were also enhanced in mushrooms treated with nanoencapsulated bitter orange (*Citrus aurantium*). CAT, SOD, and APX activities were also increased in mushrooms treated with microencapsulated carvacrol to combat oxidative damage, increasing the product’s shelf life [160]. Interestingly, the application of wax, including citral (the main EO component of lemongrass), significantly improved vitamin C content and also enhanced the activity of CAT and SOD in several oranges [161]. The high potential of including natural GRAS compounds in edible coatings of citrus fruit has been widely reported and recently reviewed [162].

## 5. Active Packaging including Bioactive Compounds

**Figure 3:**
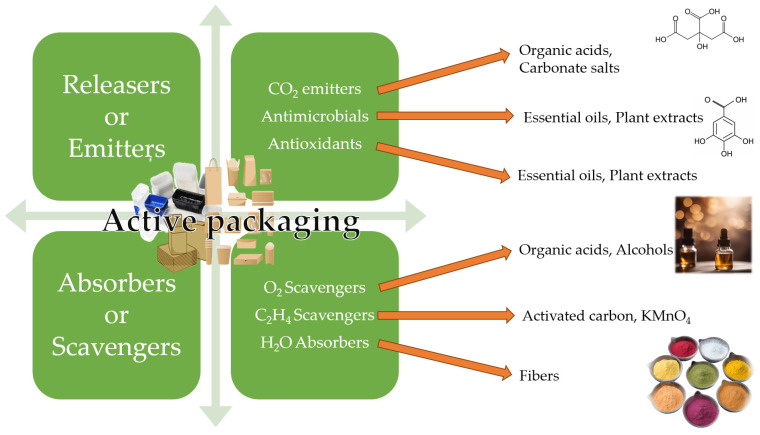
Current applications of active packaging.

The different ways to include bioactive compounds in active packaging have been summarized in Figure 3. Active packaging can interact with the product, extending shelf life, preserving quality, and even improving sensory attributes and safety. On the other hand, active packaging is intended to generate benefits in logistics and product distribution, reducing the amount of waste from the materials used, reducing the number of product costs, and improving handling and labeling, among others [163]. Active packaging is formed by two mechanisms: either by introducing the active component inside the packaging together with the product or by forming part of the packaging material itself. Active packaging is classified into two groups (Figure 3):

Absorbers or eliminators: There is a transfer of mass from the contents to the active packaging. They absorb chemical substances from inside the packaging—for example, absorbers of oxygen, water, and ethylene, among others.

Releasers or emitters: There is a transfer of mass from the active container to the contents of the container. They release chemicals into the package—for example, antioxidants, flavoring agents, antimicrobials, colorants, flavorings, and EOs.

Active packaging with encapsulated EOs has attracted great attention due to the natural origin of these compounds with high antimicrobial and antioxidant properties. Active packaging with controlled release of antimicrobial components shortens the microbial growth phase and increases the stationary phase, thus improving food safety [164]. EOs can be added directly to biodegradable polymer films, but most polymeric materials are hydrophilic, which limits the direct incorporation of these hydrophobic oils [165]. In that sense, the encapsulation of EOs into molecules with hydrophilic external surfaces (such as cyclodextrins) may overcome such limitations.

Active packaging, including nanoencapsulated citrus EO (lemon, orange, and grapefruit), reduced by 25–30% decay by *Botrytis cinerea* in strawberries, extending the product’s shelf life [166]. Literature is full of polymeric active packaging, including active components [167,168]. Nevertheless, plastic packaging must be reduced according to the European strategy for plastics (adopted in 2018) to considerably reduce their use by 2023.

Active packaging using cardboard, including encapsulated EOs within cyclodextrins, is an ecofriendly, efficient approach to extend the product’s shelf life. EOs may be encapsulated within cyclodextrins, forming inclusion complexes.

**Figure 4:**
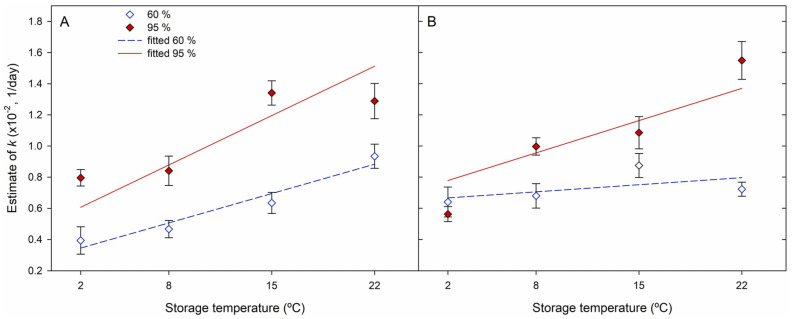
Relationship between the release rate (k, in 1/day) and the storage temperature for the carvacrol release from closed active packaging at two load levels ((**A**), 1000, and (**B**), 500 mg m^−2^ carvacrol: βCD inclusion complex) under different relative humidities (60% and 95%) (number of replicates (n) =  3  ±  standard deviation (SD)). Reprinted with permission from [63], Copyright Elsevier (2023).

The formation of the EO—CD inclusion complex, which was first described by Cramer et al. [169] in five simple steps, is a dynamic equilibrium that permits the guest to diffuse reversibly from the cyclodextrin cavity due to several variables like storage temperature and relative humidity [170]. Many hypotheses explain how temperature increases speed the release of EOs from the CD-inclusion complex: (i) the acceleration of molecular Brownian motion; (ii) the extent of wall material damage (material’s aperture widening, cracks, etc.) increases with temperature, speeding up the release of core material; and/or (iii) as the temperature rises, the energy of the gas molecules increases, increasing the percentage of activated molecules (compiled by Ren et al. [171]). Regarding the relative humidity, the rise in the number of water molecules in the environment causes imbalances in the CD-EO limits, the inclusion complex to dissociate, and finally, the release of the guest molecule, which is then replaced by water molecules [172,173]. In that sense, higher temperature and relative humidity conditions enhanced the release of the active components from the active packaging, as observed in Figure 4 [63].

## 6. Development of Clean Label Food Products Using Plant-Based Natural Additives: Integration of Natural Additives in Animal-Origin Food Products—A Study Case

In today’s society, marked by a prevalence of obesity and chronic degenerative diseases, the concept of “functional food” has gained significant traction. According to the consensus document crafted by FUFOSE (Functional Food Science in Europe), a food is deemed functional when it has been proven to have beneficial effects on specific functions in the human body beyond its typical nutritional impact. This becomes relevant for improving health and reducing the risk of disease (ILSI Europe, 1998). These foods are increasingly becoming a convenient choice, particularly for population groups prone to dietary deficiencies or individuals seeking to enhance their diet. Consequently, the food industry has invested in exploring the nexus between nutrients and diseases, aiming to introduce new products that cater to the health-conscious concerns of the current population.

In addition, the increasing demand for clean-label food mirrors consumers’ preference for more natural food products. Recent studies indicate that individuals seeking clean-label food are specifically drawn to familiar ingredients, shorter ingredient lists, and ingredients that undergo minimal processing [174].

In animal-origin products, various possibilities exist for designing potential functional and clean-label foods that either enhance the presence of beneficial compounds or limit those with potential adverse effects on consumer health, such as saturated fatty acids. Two primary strategies are employed: endogenous enrichment through modifications in animal production, including genetic and nutritional adjustments, and exogenous enrichment through the direct transformation of raw materials or the formulation of processed animal-origin products by incorporating potential functional ingredients. This exogenous enrichment involves the application of plant bioactive compounds, often derived from food industry byproducts, directly to the animal before slaughtering or during the processing and packaging stages of manufactured animal-origin products.

### PostMortem Antioxidant Strategies

Strategies employed in optimizing the composition of meat products often revolve around reformulation, aiming to reduce or eliminate harmful compounds and introduce substances with positive health implications, thereby promoting the functional nature of these derivatives. The focus of the food industry, when addressing harmful compounds, includes fat, calories, sodium nitrite, and various synthetic additives like sulfites, BHA, or BHT. These additives can pose health risks, especially for individuals consuming large amounts or those with vulnerabilities due to existing health conditions.

In the development of meat products, a range of ingredients serves diverse purposes, primarily enhancing technological properties during processing. These include colorants, flavorings, sweeteners, acidulates, seasonings and spices, emulsifiers, stabilizers, salts, phosphates, preservatives, antioxidants, humectants, and fat or salt substitutes. Notably, there has been a growing emphasis on using natural plant ingredients as a strategy in the past decade. The incorporation of these plant-based substances, both endogenously and exogenously, in animal-origin food products is seen as beneficial for health.

Incorporation of these natural substances occurs directly or as constituents of various ingredients like extracts, flours, concentrates, and homogenized forms. Numerous studies have evaluated the consequences of these processes on transformation, conservation, commercialization, and conditions of consumption. Components extensively studied in addition to functional foods include lipids, proteins, peptides, amino acids, probiotics, prebiotics or symbiotics, various antioxidants, minerals, phytosterols, phytoestrogens, and compounds such as polyphenols, soy isoflavones, or sulfurized compounds from garlic or onion, among others.

Efforts to produce animal-origin products with a healthier lipid profile involve substituting animal fat with fish or vegetable oils. This substitution results in products with lower saturated fatty acids and cholesterol content, increased amounts of MUFA and PUFA, and improvements in the Ω-6/Ω-3 ratio. Numerous studies focusing on ingredients such as olive oil [175], nuts [176], canola oil [177], grape seed EO [178], rosemary EO [179], rice fiber [177,178], linseed EO [180], or fish oil [181,182], among others, have demonstrated improvements in health properties while maintaining shelf life.

Additionally, evidence suggests that antioxidants consumed in the diet contribute to preventing oxidative damage to the body, limiting lipid oxidation in food, and reducing the risk of certain diseases, including cardiovascular diseases, certain cancers, Alzheimer’s, and cataracts [183,184].

Simultaneously, the food industry grapples with substantial waste in the form of skins, seeds, and leaves, posing environmental challenges and incurring costs for disposal. However, many fruit residues rich in phenolic compounds are being extracted and repurposed by food industries as antioxidants and antimicrobial preservatives, providing a sustainable solution to waste management.

In this context, there has been extensive research on the incorporation of polyphenols into meat and meat products. Numerous studies have explored the benefits, such as inhibiting lipid oxidation, achieved by adding grape extract to chicken meat [185], citroflavan-3-ol to lamb patties [186], cherry and blackcurrant extract leaves to pork sausages [187], kinnow rind powder to goat meat patties [188] and pomegranate powder, pomegranate seed powder. Additionally, the introduction of polyphenols to meat or meat products has shown positive effects on color [189] and sensory properties [190] without adversely impacting textural properties [191].

In recent times, considerable scientific attention has been directed towards leveraging vegetable byproducts, particularly as rich sources of carotenoids, in meat processing. Among these byproducts, those derived from tomatoes have garnered significant interest. The impact of incorporating tomato byproducts into meat quality is, in part, associated with the concurrent addition of fiber, particularly affecting textural properties. For instance, the inclusion of tomato peel in beef hamburgers not only heightened the redness of the meat but also increased its hardness simultaneously [192]. In contrast, other studies have indicated that tomato byproducts contribute to a reduction in lipid oxidation and rancidity [193].

Moreover, Robert et al. [194] undertook the reformulation of low-fat pork meat systems by incorporating 100 mg of oleuropein/kg as a byproduct from the olive oil industry. Consequently, the pork meat exhibited commendable physical stability during refrigerated storage for 14 days. The encapsulation of oleuropein proved effective in impeding the degradation of this antioxidant compound, resulting in meat with lower peroxide and malondialdehyde contents alongside a heightened antioxidant capacity.

Similarly, Martínez et al. [195] developed various meat products exogenously enriched in HXT. For example, chicken nuggets containing 750 ppm HXT from olive leaves exhibited reduced microbial growth, enhanced oxidative stability, and maintained good sensory quality for 12 months at −18 °C [195]. In addition, it compared the impact on meat derivatives of this olive tree derivative, serving as a natural source of HXT against synthetically obtained HXT.

## 7. Conclusions and Future Trends

In recent years, there has been a growing trend to replace chemical additives with ingredients rich in bioactive products of plant origin. This trend can be summarized in three stages: Stage 1 (or initial situation) is the use of food additives with different technological functions based on pure chemical compounds obtained by chemical synthesis, which significantly reduces the naturalness index of foodstuffs. Stage 2 (or intermediate situation) is the substitution of chemical additives by plant extracts rich in a certain bioactive product (these extracts can reduce the naturalness index of the food to which they are applied to a greater or lesser degree). Stage 3 (or optimal situation) is the substitution of chemical additives with food ingredients of plant origin containing a variety of bioactive compounds that do not reduce the naturalness of the foods to which they are applied. It should also be noted that Stage 3 is in line with another growing trend of using fruit and vegetable byproducts, such as skins, seeds, stems, and leaves, which are very rich in bioactive products from which Stage 3 ingredients can be obtained with nutraceutical and technological properties capable of substituting different chemical additives.

In addition, one of the issues that has also been highlighted by other authors is the fact that plant extracts and natural aromas are relatively expensive. In some cases, they are much more expensive than the chemical compounds that they want to replace. One of the solutions proposed is the development and optimization of processes for obtaining these plant bioactive compounds using biotechnology, the synthesis by biocatalyst. That is, using enzymatic or microbial fermentations and even through genetically modified or recombinant microorganisms. But this route can pose serious food safety problems that must be solved. In addition, to reduce the costs of these bioactive compounds, it is necessary to advance research work trying to optimize the extraction and purification processes from plant byproducts, which must be carried out using green technologies, avoiding the use of organic synthesis solvents producing residuals.

## Figures and Tables

**Table 1:** Anthocyanins with interest as natural plant colorants.

Anthocyanin	Color	Plant Sources
Pelargonidin	Orange	Red radish, strawberry, grape
Cyanidin	Red	Apple, red cabbage, peach, raspberry, black current, blueberry, cherry, plum, strawberry, grape
Delphinidin	Blue	Black current, blueberry, grape
Peonidin	Reddish purple	Cherry, plum, blueberry, grape
Malvidin	Violet	Blueberry, grape
Petunidin	Purple	Chokeberries, saskatoon berries, some species of grapes (e.g., grape vine, muscadine, Vitis rotundifolia)

## Data Availability

Data are contained within the article.

## Abbreviations

FNI: Food Naturalness Index; BHA: butylated hydroxyanisole; BHT: butylated hydroxytoluene; GRAS: “generally recognized as safe”; EO: essential oil; HXT: hydroxytyrosol; EVOO: extra virgin olive oil; OH: hydroxyl group; AH: antioxidant compound (AH); R•: free radical; A•: stable radical; DDMP: (2,3-dihydro-2,5-dihydroxy-6-methyl-4H-pyran-4-one)); PPO: polyphenol oxidase; POD: polyphenol peroxidase; PAL: phenylalanine ammonia-lyase; PE: pectinesterases; PG: polygalacturonase; LOX: lipoxygenase; ACC: l-aminocyclopropane-1-carboxylic acid (ACC); ACO: ACC oxidase; ACS: ACC synthase; SOD: superoxide dismutase; CAT: catalase; APX: ascorbate peroxidase; GR: glutathione reductase; CD: cyclodextrin; MUFA: monounsaturated fatty acids; PUFA: polyunsaturated fatty acids.
